# Supplementary material for: Giotto: a toolbox for integrative analysis and visualization of spatial expression data
Source: Genome Biol. 2021 Mar 8;22:78. doi: 10.1186/s13059-021-02286-2 (PMC7938609; doi:10.1186/s13059-021-02286-2)
Supplement: Supplementary file 2 — Additional file 1. Supplementary Figures. Supplementary figures and legends. [file 13059_2021_2286_MOESM1_ESM.docx]

**Supplementary Figures**

**
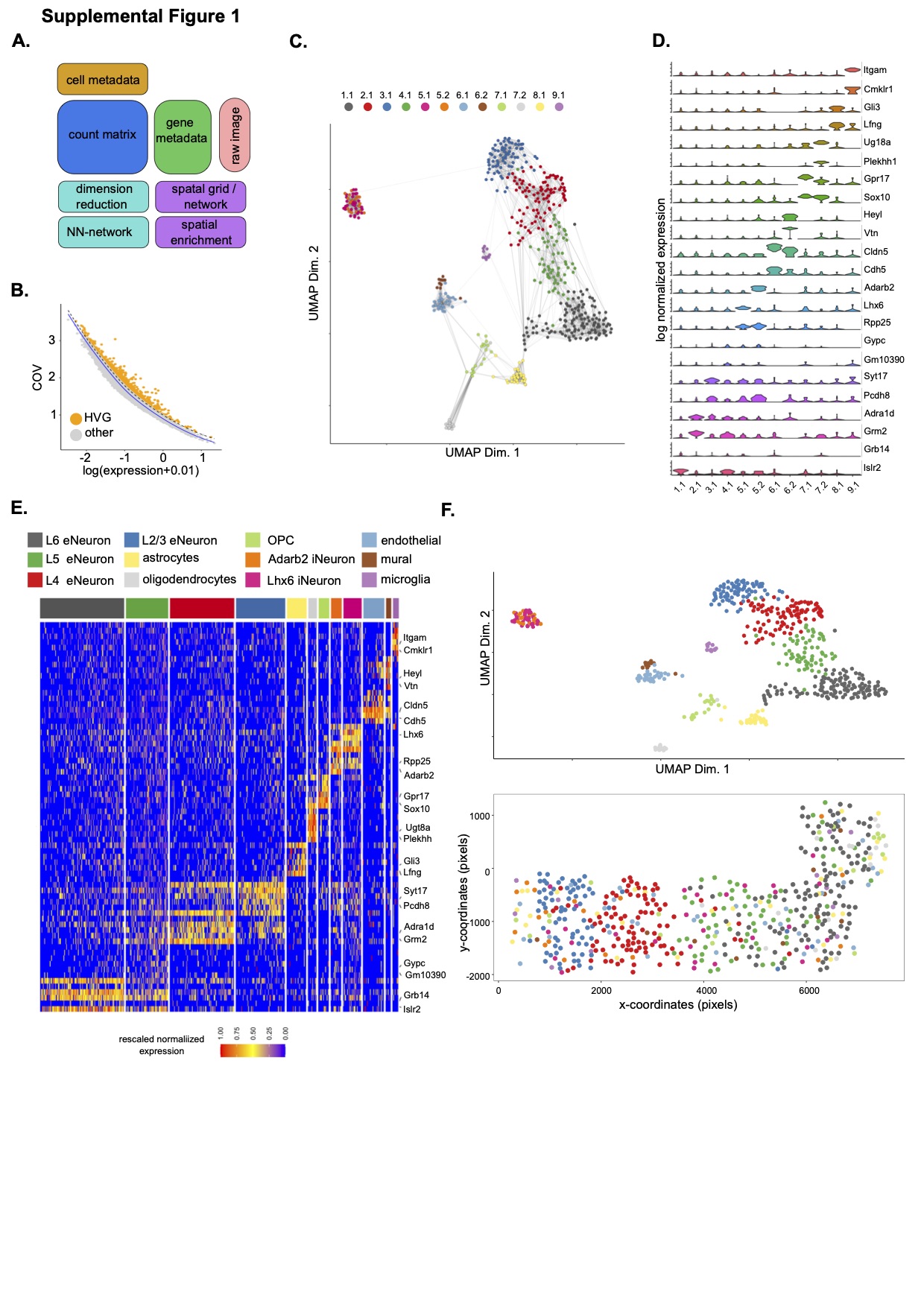
**

**Figure S1. Detailed cell-type identification.
A.** Schematic representation of the Giotto S4 object showing the primary slots for accessing and storing data and analyses. Nearest-neighbor (NN) network in expression space. **B.** Scatterplot showing the coefficient of variation versus average expression level for all genes in the seqFISH+ mouse somatosensory cortex dataset. Highly variable genes (HVG) exhibit more than expected variation compared to predicted scores based on loess regression (blue line). **C.** Dimension reduction visualization (first 2 dimensions of UMAP) of the seqFISH+ somatosensory cortex dataset. Colors indicate the different clusters identified with the Leiden community detection approach. **D.** Violinplot highlighting cluster specific marker genes identified with the Gini algorithm for the clusters identified in **C. E.** Heatmap showing clusters form **C** and **E** annotated for cell type based on specific marker genes. **F.** Visualization of the seqFISH+ somatosensory cortex data in both expression (top) and physical space (bottom), distance unit = 1 pixel (1 pixel ≈ 103 nm).

**
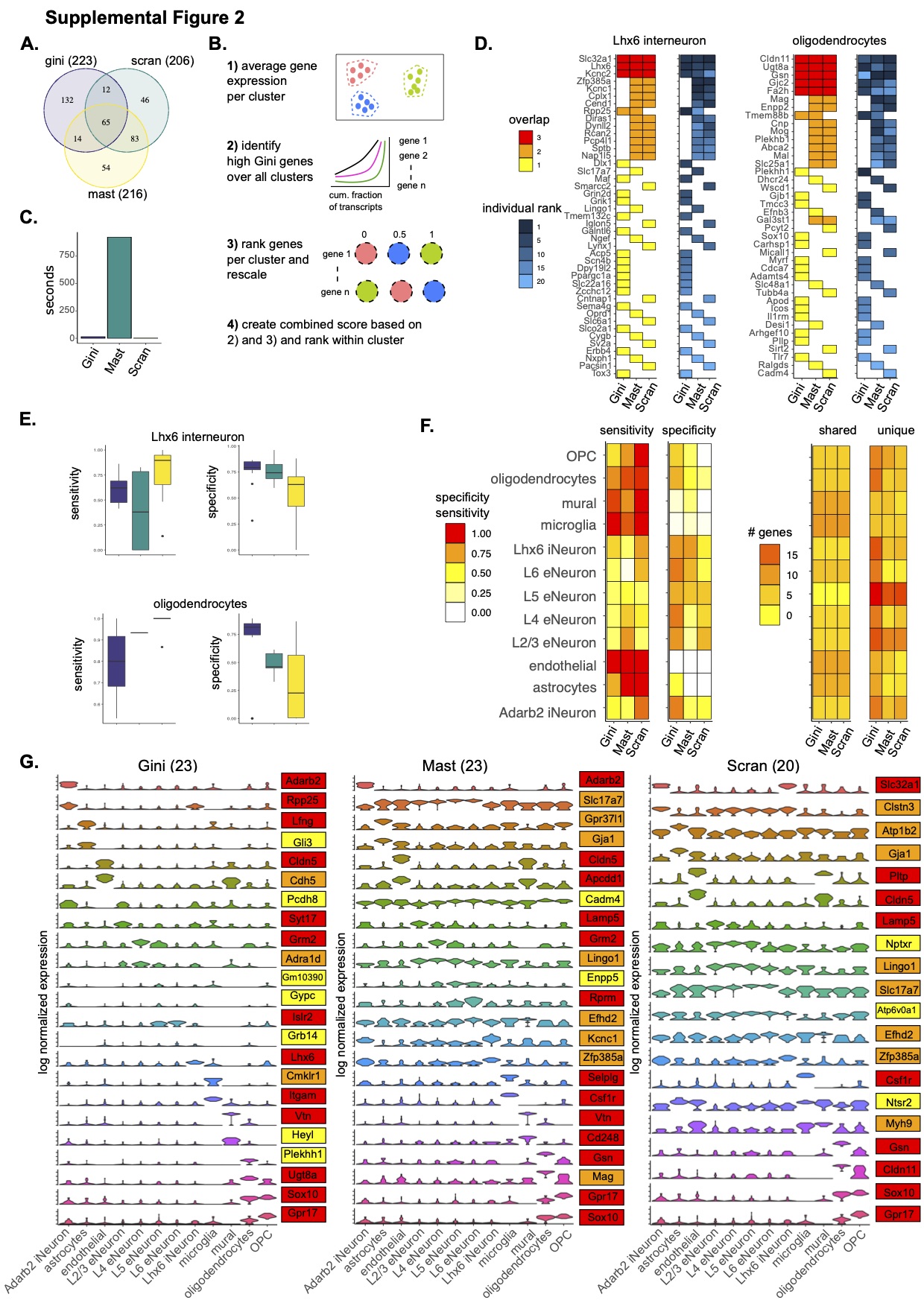
Figure S2. Evaluation of the Gini-based marker gene detection method.
A.** Venn diagram depicting the overlap of the top 20 identified marker genes for all 12 cell types (see **Fig. S1E**) in the seqFISH+ somatosensory cortex dataset for all 3 methods provided by Giotto. **B.** Schematic explanation of the Gini-based method to identify cell type specific marker genes. **C**. Run time comparison for the 3 depicted marker gene detection methods. **D.** Heatmap showing the combined top 20 genes identified by the 3 different marker gene detection methods in Lhx6+ interneuronal cells (left) or oligodendrocytes (right). The yellow, orange and red colors represent the number of times a gene was detected in all 3 methods. The blue color gradient shows the individual ranking for the top 20 genes in each method. Dark means higher prioritization. **E.** Boxplots showing the difference in sensitivity or specificity for the genes that are only prioritized (top 20) by one method, but not by others for the Lhx6+ interneuronal cells (top) and oligodendrocyte (bottom) populations. **F.** Heatmaps generalizing the analysis in **D** and **E** to all cell types. Heatmaps on the left show the sensitivity and specificity differences for the method specific marker genes and heatmaps on the right show the overlap results for the top 20 genes. **G.** Violinplots showing the top 2 marker genes per cell type for each marker gene detection method. Colors on the left indicate the overlap with other methods, similar as in **D**.

**
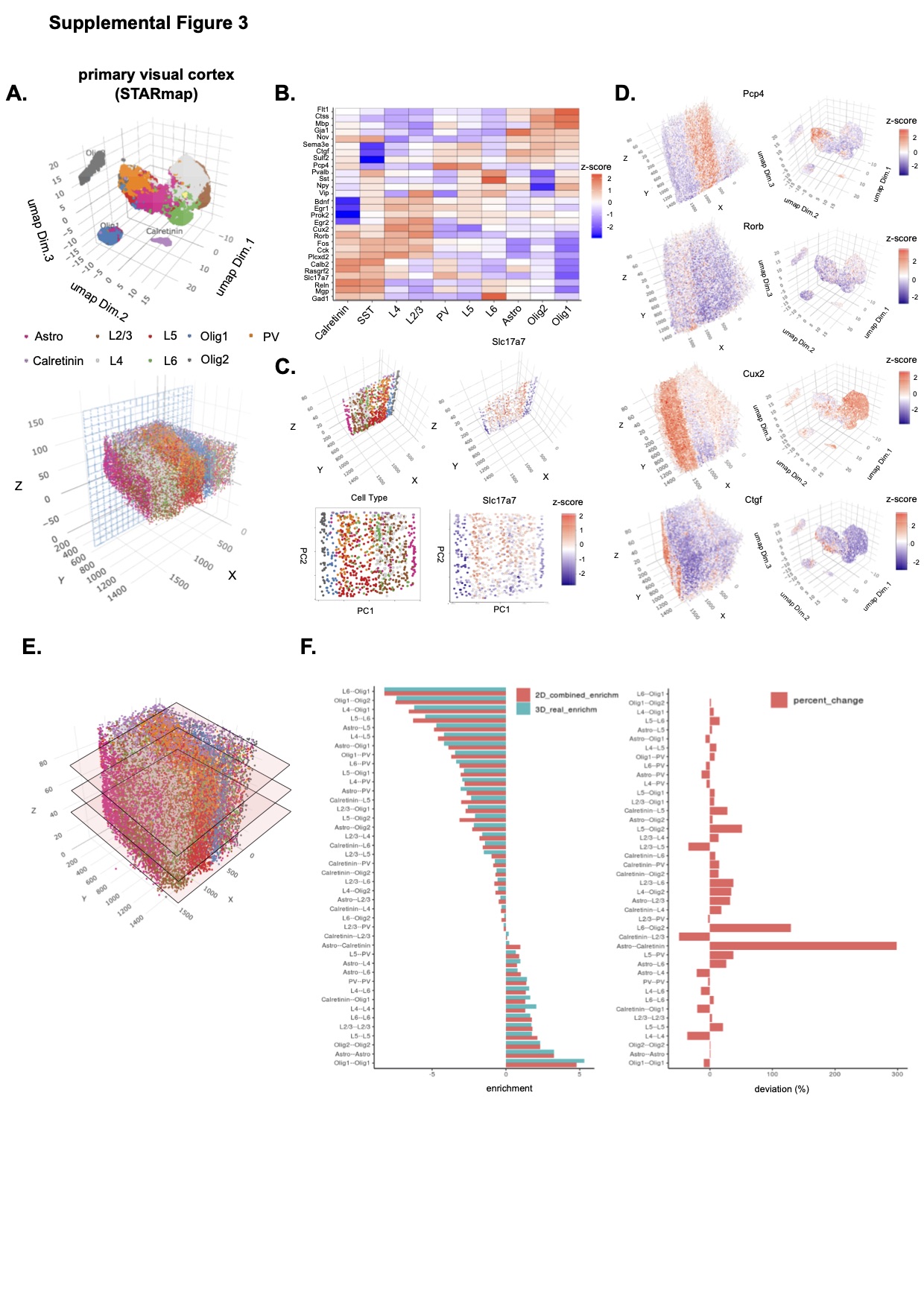
**

**Figure S3. Analysis and visualization of 3D dataset.**

**A-D.** Analysis of the mouse visual cortex STARmap dataset, which is a 3D sample (distance unit = 1 µm)**. A.** Visualization of the identified cell types in both expression (top) and physical (bottom) space. **B.** Heatmap showing the cell-type specific marker genes**.** **C.** Visualization of a subset of cells in a user-defined plane (blue grid in **A**) in 3D (top) or 2D (bottom). Colors represent the identified cell types (left) or expression level of any selected gene (right). **D.** Overlay of expression values for the indicated genes in both expression and physical space for the whole 3D dataset. **E.** Schematic representation of the generation of multiple virtual slices (n = 3) along the z-axis of the STARmap dataset. The z-axis is rescaled compared to **A**. **F.** Barplot (left) depicting the ratio of observed over expected frequencies of pairwise interacting cell types for the full 3D STARmap dataset (red) and the combined results over 7 consecutive 2D slices along the z-axis (green). Barplot (right) showing the percent deviation of the relative difference between the 3D and 2D results.


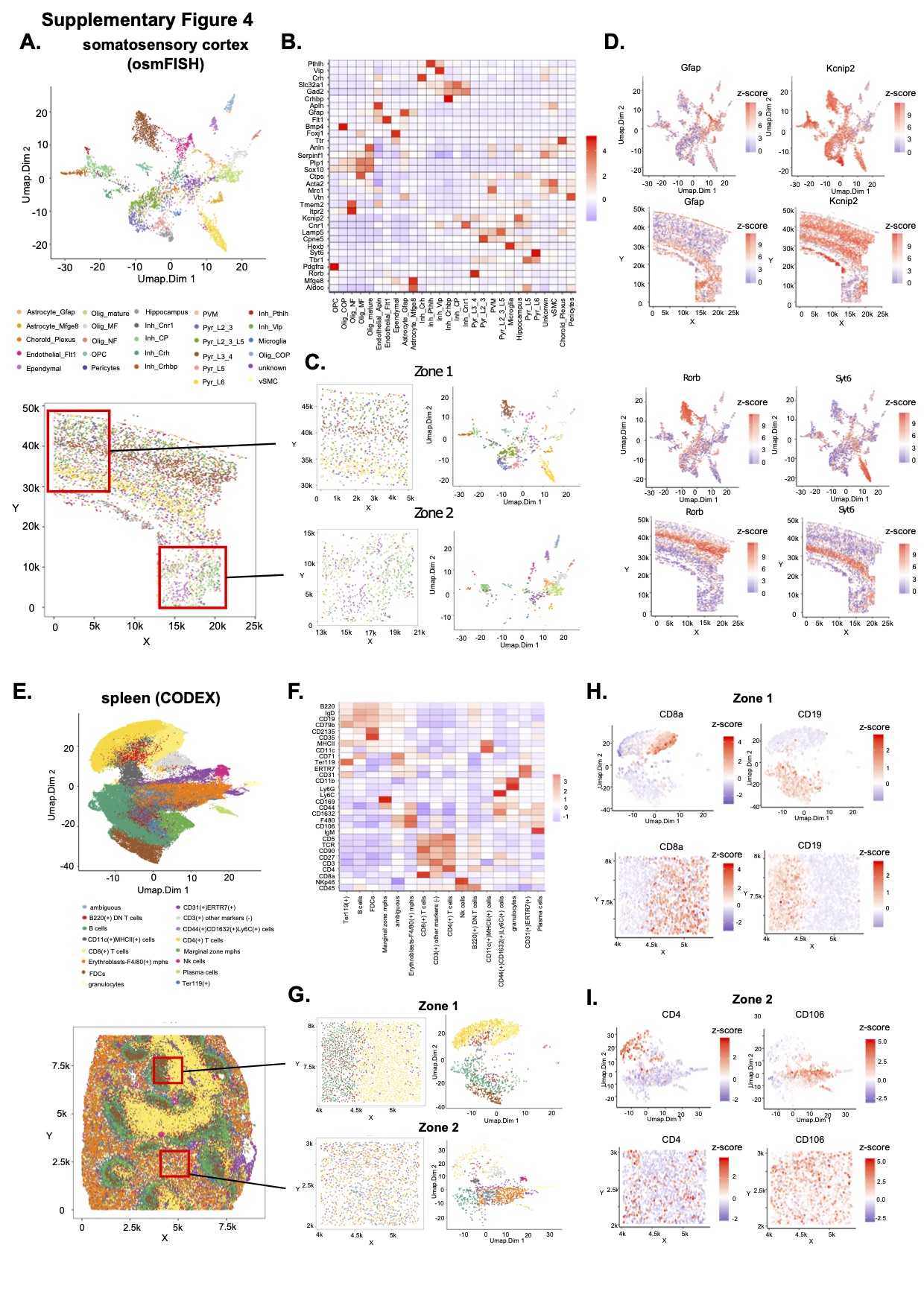


**Figure S4. Analysis and visualization of the mouse cortex osmFISH and spleen CODEX datasets**

**A-D.** Analysis of the mouse somatosensory cortex osmFISH dataset (distance unit = 1 pixel, 1 pixel ≈ 67 nm). **A.** Visualization of the identified cell types in both expression (top) and physical (bottom) space. **B.** Heatmap showing the cell-type specific marker genes**. C.** Zoomed-in view of the two highlighted subregions (indicated by red squares) in **A. D.** Overlay of expression values for the indicated genes in both expression and physical space. **E-I** Analysis of the mouse spleen CODEX dataset**. E.** Visualization of the identified cell types in both expression (top) and physical (bottom) space (distance unit = 1 pixel, 1 pixel ≈ 188 nm). **F.** Heatmap showing the cell-type specific marker genes**. G.** Zoomed-in view of the two highlighted subregions (indicated by red squares) in **E. H-I.** Overlay of expression values for the indicated genes in both expression and physical space for zone 1 (**H**) or zone 2 (**I**).


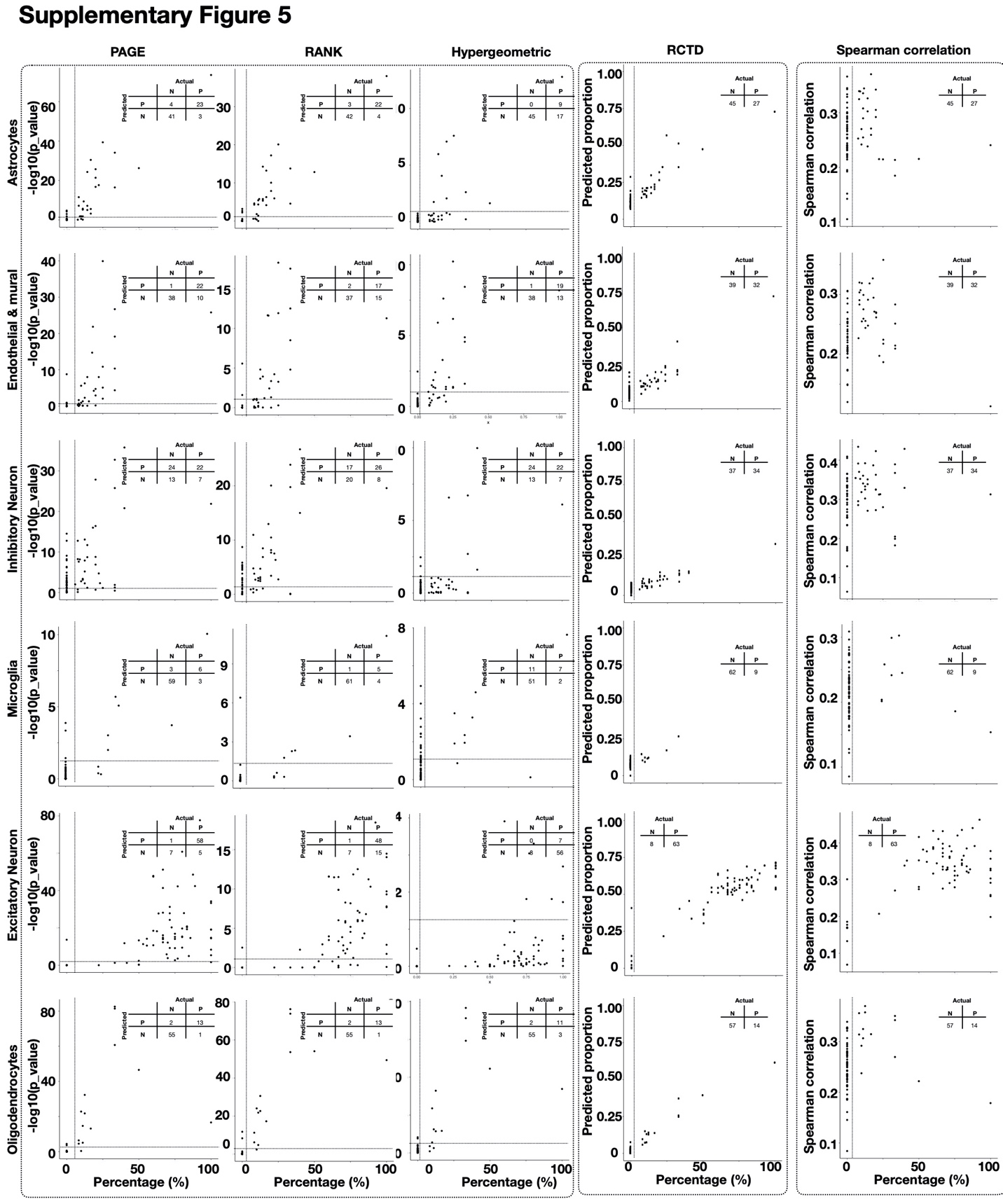


**Figure S5.** **Scatter plots showing the relationship between inferred cell type enrichment scores (y-axis) and observed cell-type frequency (x-axis).**

Different cell type enrichment methods are applied to the simulated coarse-resolution spatial transcriptomic dataset. For PAGE, RANK, and hypergeometric analyses, the enrichment scores are converted to p-values as described in the Methods section. The horizon dash lines indicate the cutoff of p-value = 0.05. For RCTD and Spearman correlation, there is no conventional cutoff. The vertical dash lines indicate the boundary between present and absent squares.


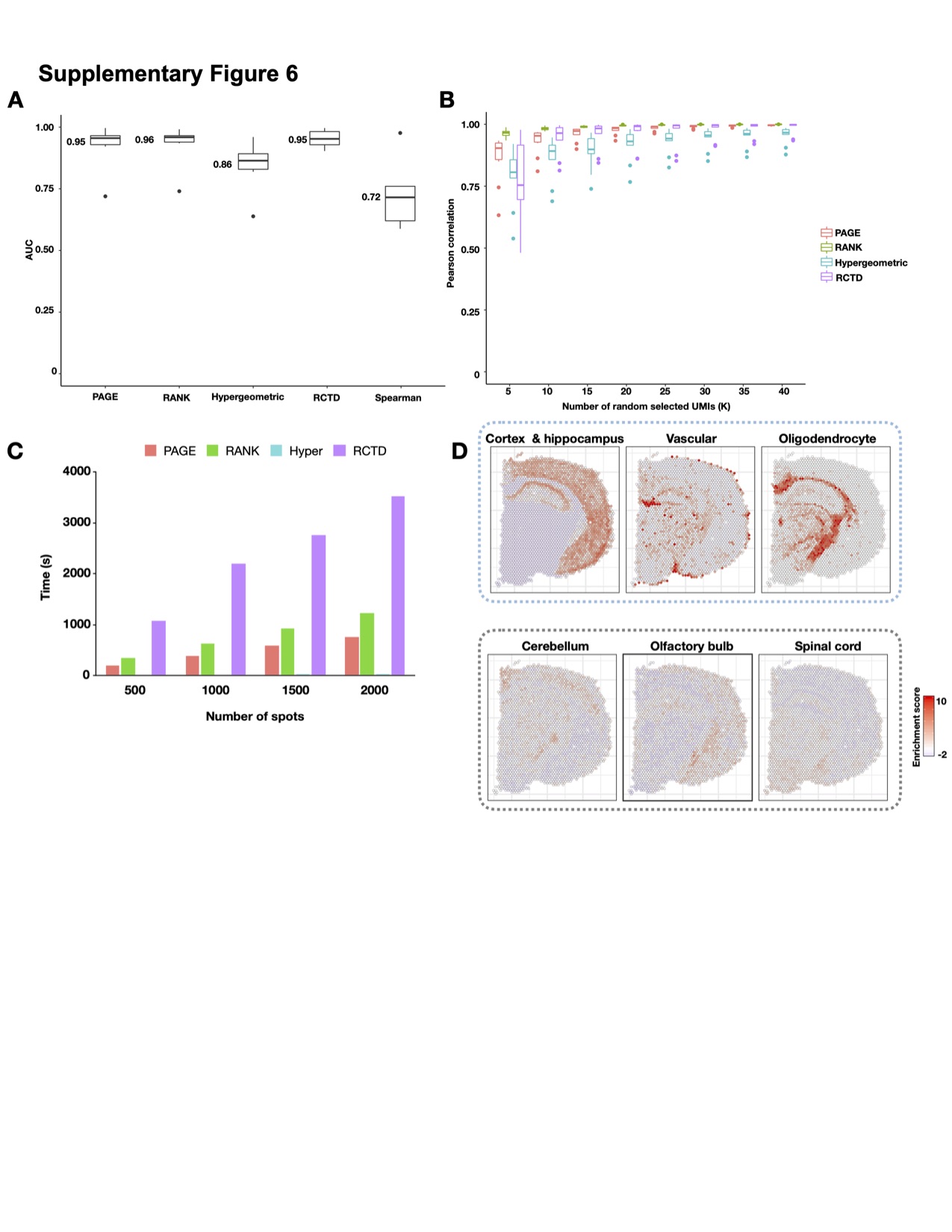


**Figure S6. Evaluation of the performance of different cell type enrichment analysis methods.**

**A-B.** Different cell type enrichment methods are applied to the simulated coarse-resolution spatial transcriptomic dataset. **A.** The accuracy of using the enrichment scores from each method to predict the presence of a cell type in a square is quantified by the corresponding AUC scores. **B.** Robustness analysis of the four methods associated with high AUC scores. Each method is applied to analyze random subsamples with varying sequencing depth (as quantified by detected UMI numbers). The agreement between these and the original results is quantified by the Pearson correlation coefficient. **C.** Barplot showing the computer running time of each method for various numbers of spots. **D.** Cell type enrichment analysis of the mouse brain Visium dataset. The enrichment score distributions corresponding to various cell types are displayed. Notice that the enrichment score values for the three negative control cell types (indicated as the group on the right) are low in general. The distance unit is 1 pixel (1 pixel ≈ 1.46 µm).

~~
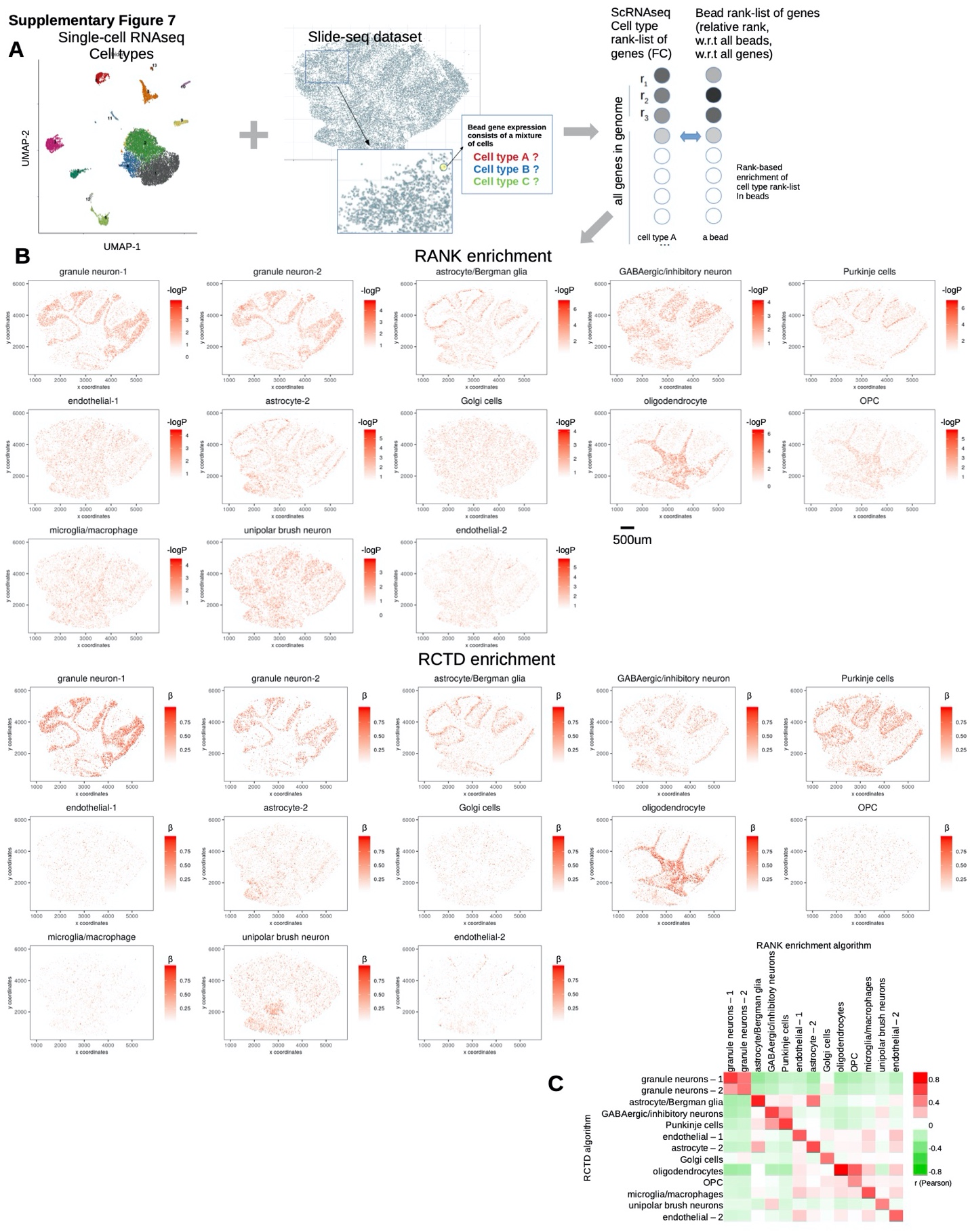
~~

**Figure S7. Cell-type enrichment analysis of the mouse cerebellum Slide-seq dataset.**

**A.** Schematic illustrating the RANK procedure for cell type enrichment analysis (see Methods for details). **B.** Comparison of using RANK (top) RCTD (bottom) to infer cell-type distributions. For the RANK method, the enrichment scores are converted to -log(p-values) (abbreviated by -logP). For the RCTD, the estimated proportion (β) is shown. **C.** Heatmap showing the Pearson correlation coefficients (r) between the spatial patterns inferred by the two methods.


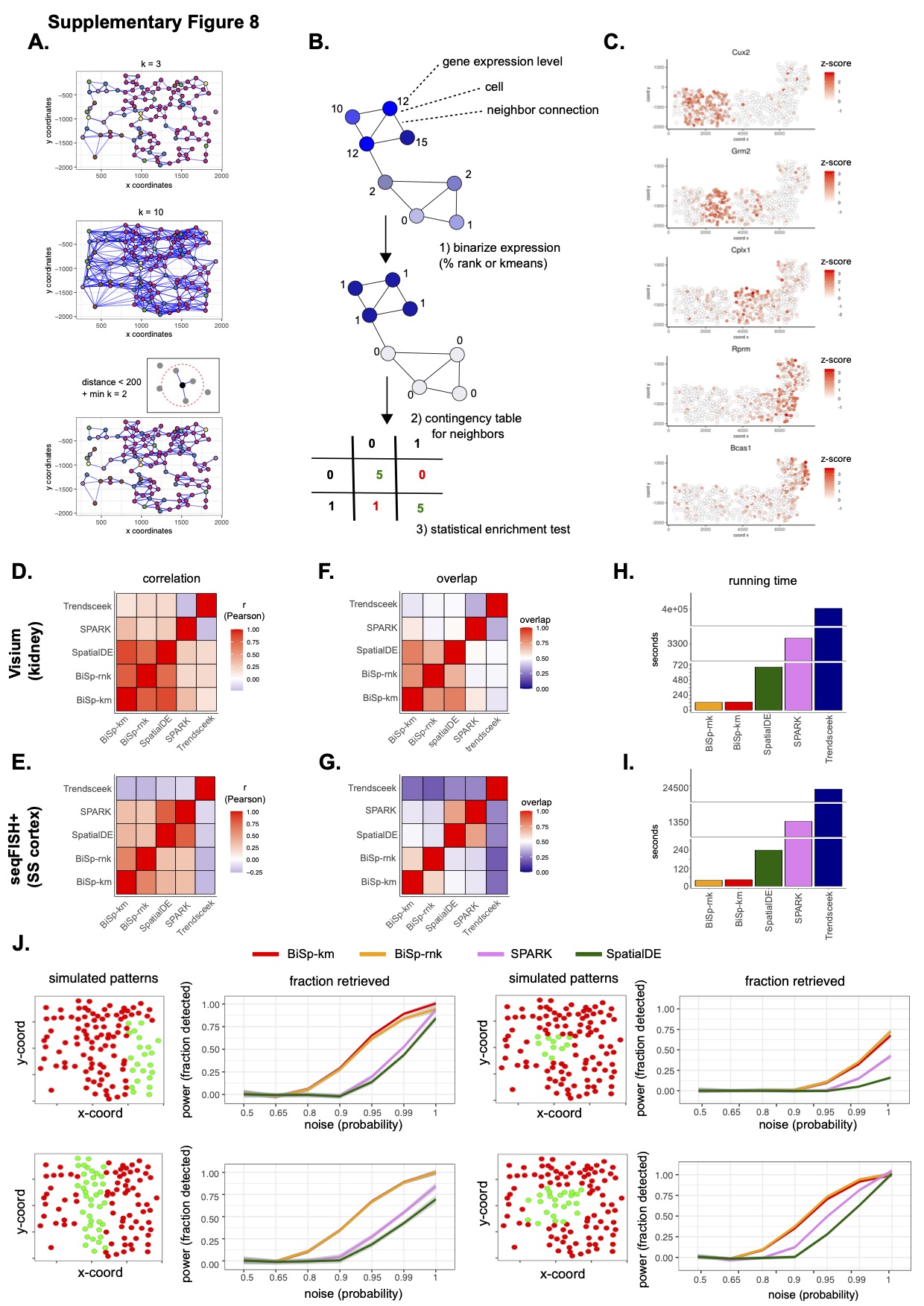


**Figure S8. Benchmarking of spatial gene detection methods.**

**A.** Visualization of spatial networks with different neighbors (k) and/or distance from the cell centroid parameters. **B.** Schematic representation of the BinSpect method to identify spatial genes based on increased observation of high binarized expression between neighboring cells within the spatial network (see **Methods** for details). **C.** Examples of spatial genes along the multi-layered seqFISH+ mouse somatosensory cortex dataset, the distance unit = 1 pixel (1 pixel ≈ 103 nm). The outer layers are on the left, while more inner layers are on the right. **D-E.** Systematic comparison of spatial gene detection methods (BinSpect-kmeans = BiSp-km, BinSpect-rank = BiSp-rnk) in the seqFISH+ somatosensory cortex dataset (**D, F and H**) and Visium mouse kidney dataset (**E, G and I**). Heatmaps with pairwise correlation (Spearman) scores for the overlapping genes between two methods (**D** and **E**). Heatmaps depicting the number of overlapping genes between two methods (**F** and **G**). Barplots depicting the run time for the different methods as implemented in Giotto Analyzer (**H** and **I**). **J.** Spatial gene detection simulation results. Different simulated patterns are show on the left (green = pattern, red = remaining cell locations) and corresponding spatial gene detection results are show on the right. A loess smoothened curve is shown for each method depicting the average fraction of retrieved genes (y-axis) at different noise levels (x-axis). Noise is controlled by a probability parameter (Pr), with 0.5 = random pattern and 1 = strong pattern.


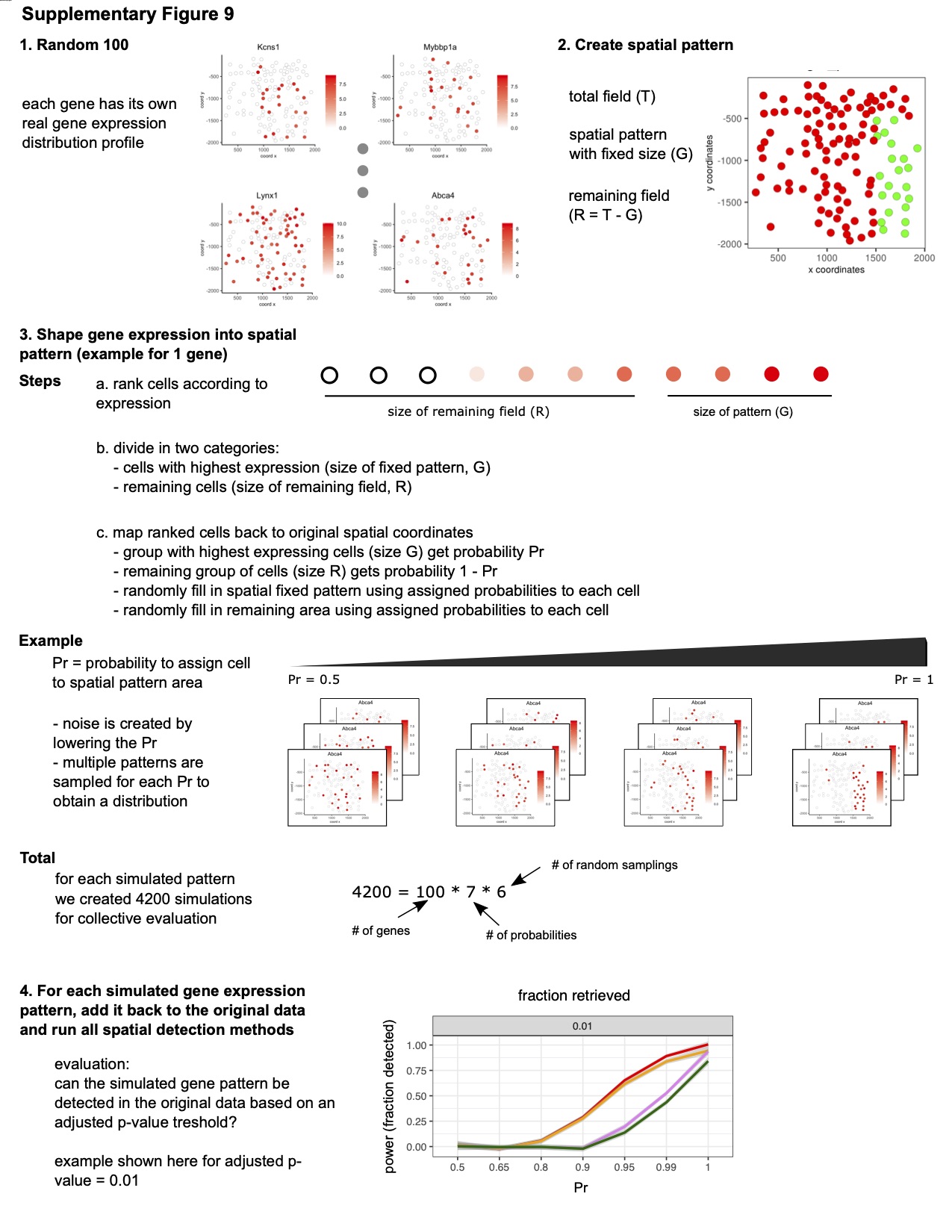


**Figure S9. A schematic of the spatial pattern simulation strategy**

Schematic representation of the simulation strategy used to quantitatively compare the different spatial gene detection methods (see **Methods** and **Additional file 3: Notes** for details).

**
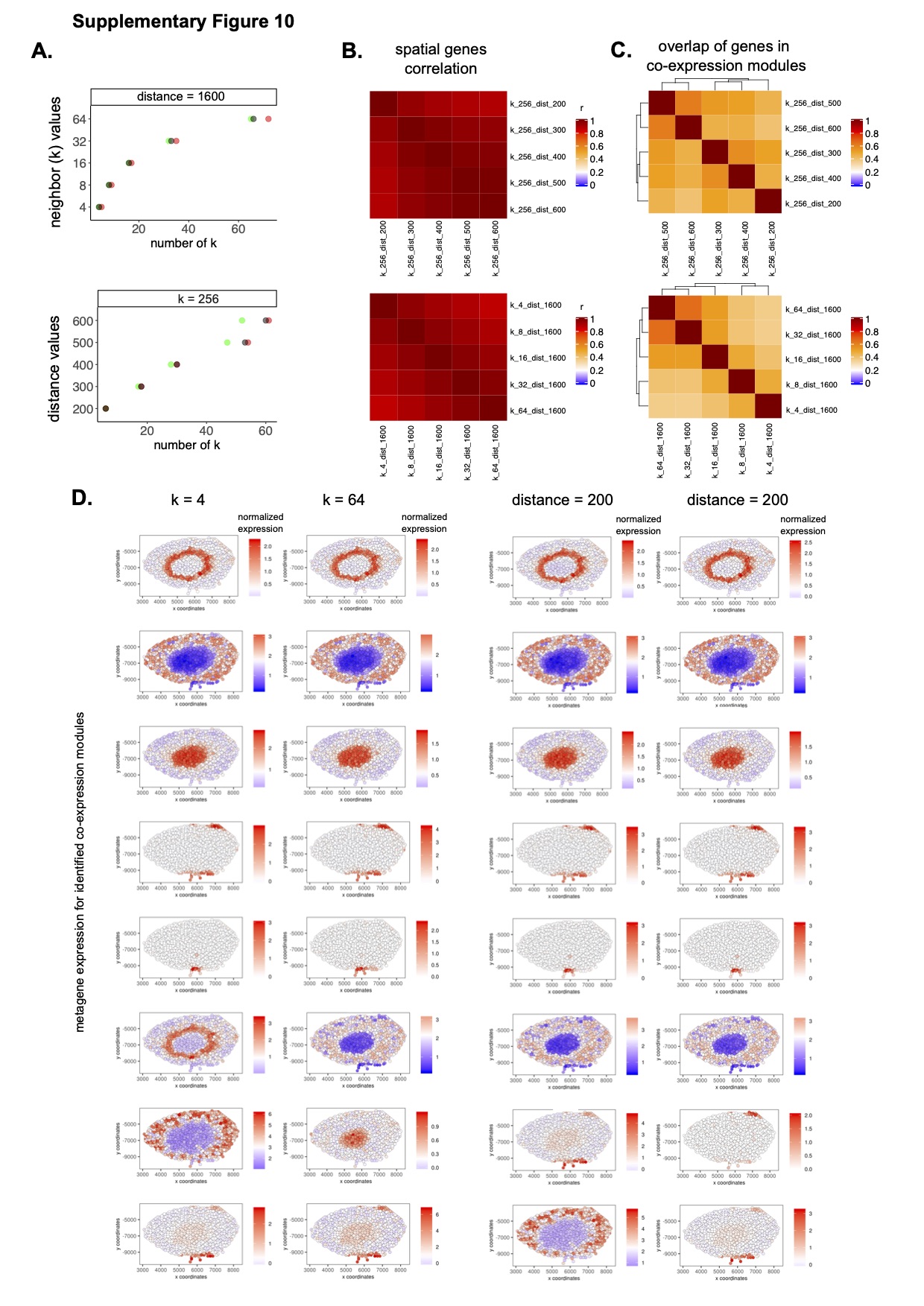
**

**Figure S10. Effect of spatial network parameters on spatial pattern detection**

**A.** Scatterplot depicting the average number of neighbors (k) for each spot in the Visium kidney dataset for different values of the radial distance (top) or k (bottom) parameter. When changing the k parameter, a large distance parameter (= 1600 pixels; 1 pixel = 1.46 µm) was fixed. When changing the radial distance parameter, a large k (= 256) was fixed. The green and red dots represent the 25^th^ and 75^th^ percentile. **B.** Heatmaps showing the (Spearman) correlation scores for all the identified spatial genes in the different conditions of **A**. **C.** Heatmaps showing the adjusted rand-index scores for the genes within the identified spatial gene co-expression modules for all the different conditions in **A**. **D.** Qualitative visualization of the metagene expression values associated with the identified spatial gene co-expression modules (k modules = 8) for the most different values in **A**. The distance unit is 1 pixel (1 pixel ≈ 1.46 µm).


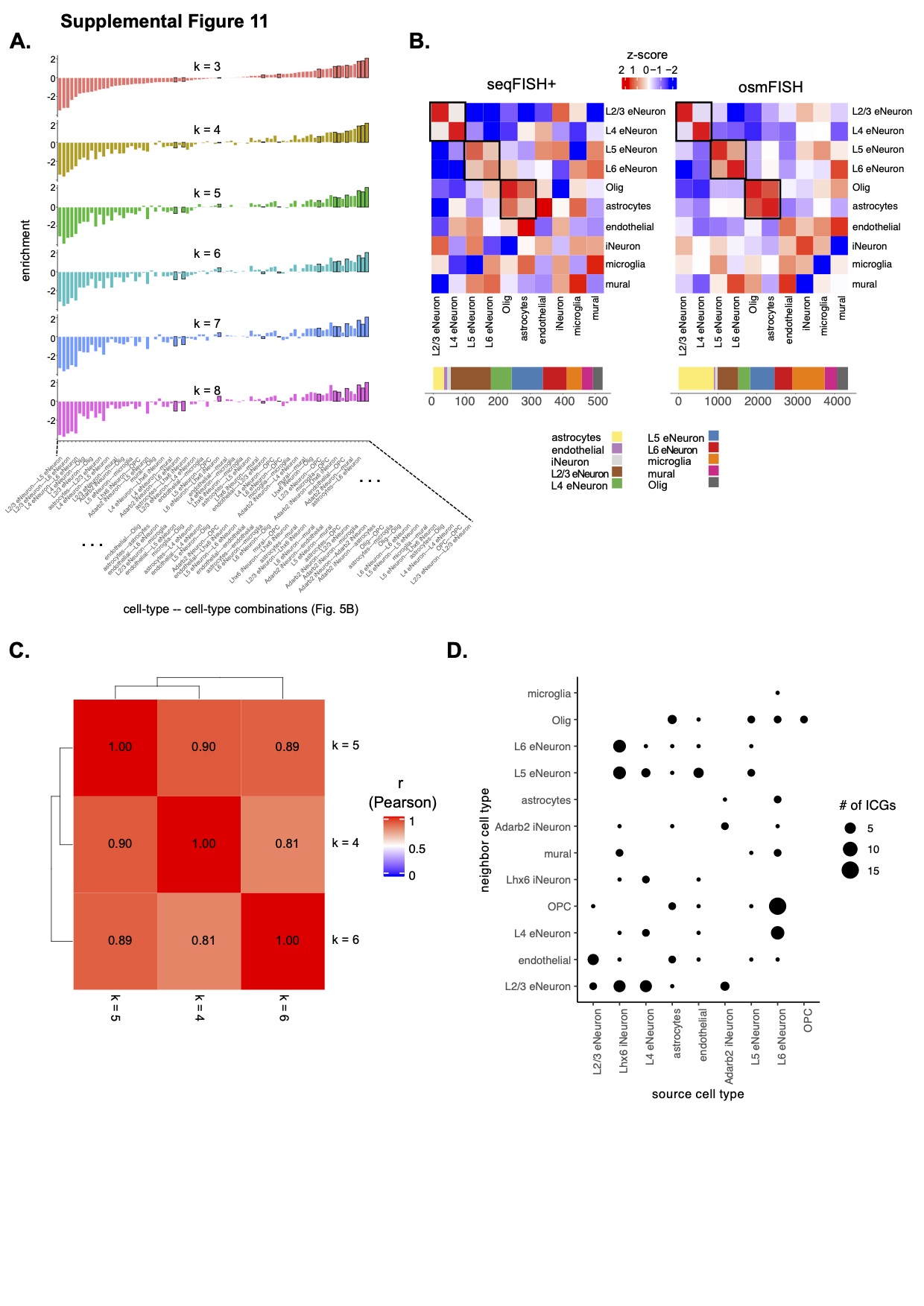


**Figure S11. Effect of spatial network parameters on cell neighborhood analyses**

**A.** Barplots depicting the ratio of observed over expected frequencies of pairwise interacting cell types within the seqFISH+ somatosensory cortex dataset for spatial networks with a different neighbor (k) parameter. Homo-typic (same two cell types) interactions display a tick border region, while heterotypic (two different cell types) interactions do not. **B.** Heatmap showing the ratio of observed over expected frequencies of pairwise interacting cell types within the simplified (see methods) seqFISH+ (left) and osmFISH (right) somatosensory cortex datasets. Enriched or depleted interactions are depicted on a scale between red and blue, respectively (top). Barplots depicting the corresponding cell-type distribution and numbers for each dataset (bottom). **C.** Heatmap showing the pairwise correlations for the significance scores of the top 200 ligand-receptor pairs for spatial networks with a different number of neighbors (k) (see **Additional file 3: Notes**).**D.** Dotplot showing the total number of interaction changed genes (ICG), which are genes that are differentially expressed in the source cell type (x-axis) due to spatial interactions with another neighbor cell type (y-axis).

~~
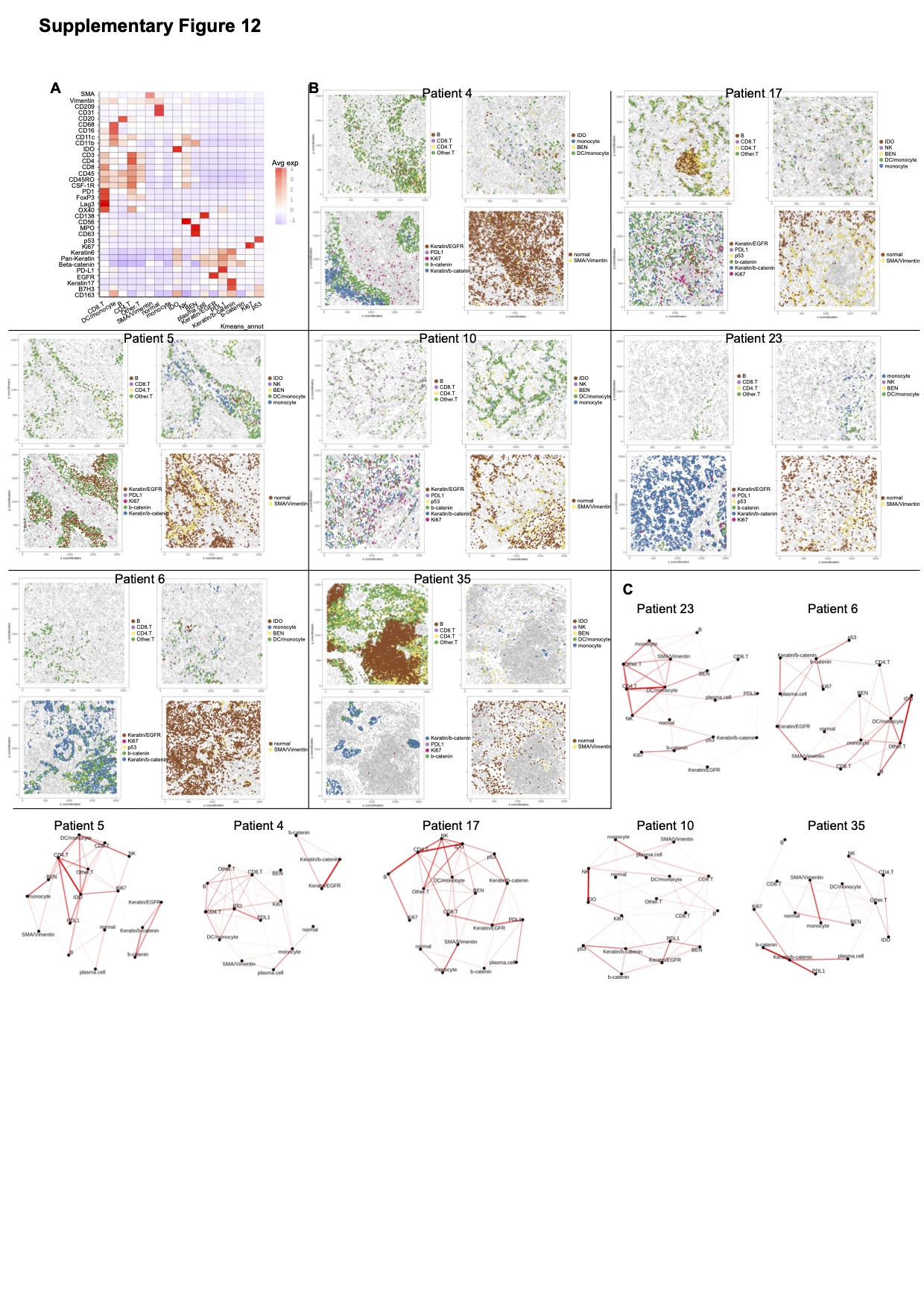
~~

**Figure S12. Analysis of MIBI triple negative breast cancer dataset.**

**A.** K-means clusters of 197,678 cells over a 34-protein marker panel. **B.** The cell-type landscape of the tumor, immune, and normal cells in 7 representative patients are delineated by black-outlined square boxes (distance unit = 1 pixel, 1 pixel ≈ 440 nm). Each square box (representing one patient) is made up of 4 panels each showing a subset of the cell populations from the same patient sample. The 7 representative patients can be roughly divided into compartmentalized spatial arrangement of cell populations (such as Patients 4, 5), and mixed arrangement (such as Patients 10 and 17). **C.** Cell type-cell type interaction network reveals differences between patients (see **Additional file 3: Notes**).

**
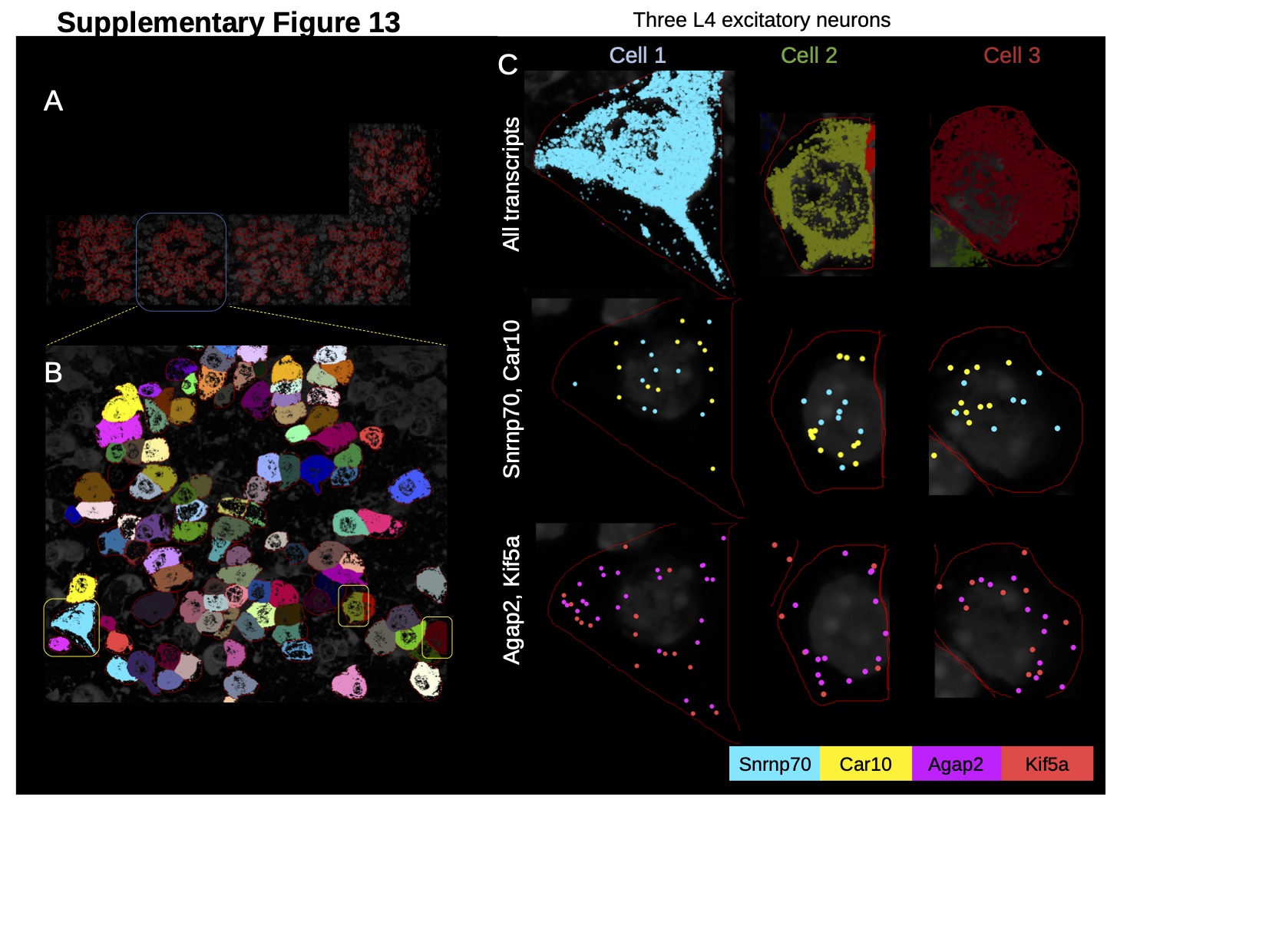
Figure S13. Visualization of transcript subcellular localization patterns.**

**A.** Nissl staining images for the mouse somatosensory cortex region obtained from seqFISH+ data. Cell boundaries are indicated by red outlines. **B.** Zoomed-in view of a selected region (indicated by the blue box in **A**.) showing the spatial locations of all the transcripts that are mapped to any segmented cell. Each dot represents an individual transcript. Colors indicate different cell types. **C.** Zoomed-in view of selected cells (indicated by the yellow boxes in **B**). Giotto Viewer can be used to visualize either all transcripts (top row) or those from selected genes (middle and bottom rows).


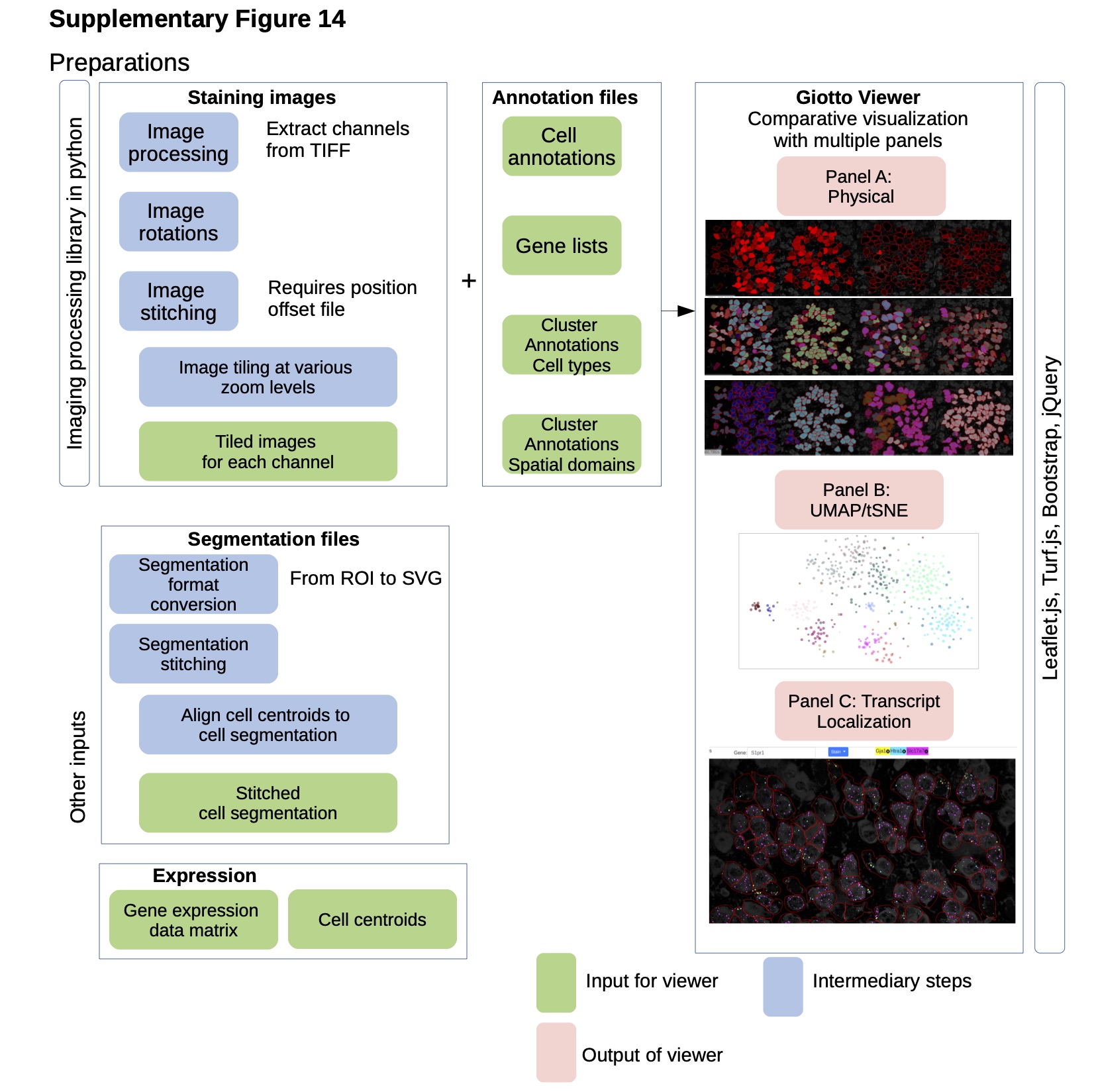


**Figure S14. Technical overview of the Giotto Viewer module.**

The different components of the Giotto Viewer setup include staining images, cell segmentation files, gene expression, and annotation files. Some of these inputs are optional. The annotation files are generated from Giotto Analyzer. The layout of the panels can be customized by the user.
