# Supplementary material for: Giotto: a toolbox for integrative analysis and visualization of spatial expression data
Source: Genome Biol. 2021 Mar 8;22:78. doi: 10.1186/s13059-021-02286-2 (PMC7938609; doi:10.1186/s13059-021-02286-2)
Supplement: Supplementary file 3 — Additional file 2: Table S1. Table of different technologies and datasets that were analyzed with Giotto. [file 13059_2021_2286_MOESM2_ESM.docx]

**Table S1**: Description of the spatial expression datasets analyzed in this paper.

**abbreviations:**

*smFISH* single-molecule fluoresence in situ hybridization

*RCA* rolling circle amplification

*IF* immune fluorescence

*MS* mass spectometry

*PDAC* pancreatic ductal adenocarcinoma

*TNBC* triple negative breast cancer

NA not available

* at spot level

** at bead level
