# Supplementary material for: Giotto: a toolbox for integrative analysis and visualization of spatial expression data
Source: Genome Biol. 2021 Mar 8;22:78. doi: 10.1186/s13059-021-02286-2 (PMC7938609; doi:10.1186/s13059-021-02286-2)
Supplement: Supplementary file 4 — Additional file 3. Supplementary Notes. Supplementary notes and additional information about data analysis with Giotto. [file 13059_2021_2286_MOESM3_ESM.docx]

**Supplementary Notes**

**Datasets**

*seqFISH+ mouse somatosensory cortex*

The seqFISH+ mouse somatosensory cortex dataset was provided by the Cai lab and published in [(Eng et al. 2019)](https://paperpile.com/c/zehMrt/fTsh). The dataset consisted of 7 fields of views, which were first stitched together with the command *stitchFieldCoordinates.* Then we only selected the first 5 field of views that overlapped with the anatomical cortex region and normalized the cells using the default settings with the command *normalizeGiotto*. To cluster cells we first computed highly variable genes with the command calculateHVG and the parameter method = ‘cov_loess’. Next, highly variable genes that were detected in more than 4% of cells and had an average mean log-normalized expression greater than 0.5 were used to run PCA with *runPCA* with parameter scale_unit = F. The first 15 PCs were then used as input for the command *runUMAP* to perform further nonlinear dimensionality reduction with UMAP. A shared nearest neighbour (sNN) network was created with *createNearestNetwork* on the PCA space with parameters: dimensions_to_use = 1:15 and k = 15. Finally, to detect clusters within this sNN network, Leiden based clustering was applied with the command *doLeidenCluster* and resolution 0.4. In a subsequent round we performed targeted subclustering of 3 clusters with *doLeidenSubCluster*. Cluster specific marker genes were identified and used to identify coarse or refined cell types, on the first and second round of clustering, respectively. The coarse cell type clusters were annotated as microglia, oligodendrocytes, endothelial cells, astrocytes, interneurons (iNeuron) and layer-specific excitatory neurons (L2/3, L4, L5 and L6 eNeurons). For the refined cell types, the endothelial cells were further divided into endothelial cells and mural cells, the interneurons contained both Lhx6 and Adarb2 positive interneurons, while the oligodendrocyte were further dissected into oligodendrocyte precursors and more mature oligodendrocytes. Different spatial grids or spatial networks were created with *createSpatialGrid*, *createSpatialNetwork* or *createDelaunayNetwork* depending on the analysis and as described here and in the main text.

To identify HMRF-based spatial domains, spatially coherent genes were first computed with the *binSpect (kmeans)* function using the default settings, followed by the creation of a Delaunay network with the function *createDelaunayNetwork*. Then the obtained Delaunay network and the top 100 ranked spatial were input into the function *doHMRF* to identify spatial domains on the scaled expression matrix. The number of domains (k) was set to 9. 3 different beta values were tested (28, 30 and 32) and beta = 28 was selected for visualization. The Delaunay network was also used as the starting point for the cell neighbourhood analysis. The *cellProximityEnrichment* function was used to identify cell-type/cell-type interaction enrichment with parameters ‘number_of_simulations = 2000’ and the results were visualized by using the function *cellProximityNetwork*. To visualize the effect of different spatial network neighbors (k) (see benchmarking results section), we first created different spatial networks with createSpatialNetwork and then used the function *cellProximityBarplot*. To compute cell-cell communication scores based on ligand-receptor pair expression we used the functions *exprCellCellcom* and *spatCellCellcom.* For the latter, we used the default spatial network generated by *createSpatialNetwork*. The results from these two analyses were plotted against each other using a heatmap with the function *plotRankSpatvsExpr*. To visualize predictive power of the spatially unaware results, we used the function *plotRecovery*. Selected ligand-receptor pairs (FDR ≤ 0.01, |log2 fold-change| > 0.25, number of interacting cells ≥ 4) were visualized in a dotplot using the function *plotCCcomDotplot.* To identify individual genes whose expression levels changed duo to spatial interactions we used the function *findICG* with the Delaunay spatial network and 2000 permutations. These were further filtered with the function *filterICG* with parameter setting ‘min_cells = 5, min_int_cells = 5, min_fdr = 0.001, min_log2_fc = 0.5 and min_spat_diff = 0.25) and the results were visualized in a dotplot with *plotICG* and parameter ‘method = “dotplot” ‘.

*osmFISH mouse somatosensory cortex*

The osmFISH dataset and accompanying information was downloaded from <https://linnarssonlab.org/osmFISH/> in companion with [(Codeluppi et al. 2018)](https://paperpile.com/c/zehMrt/PbM8t). After creating a Giotto object from the spatial cell coordinates and gene expression matrix with *createGiottoObject*, we used the *filterGiotto* command to remove cells with less than 10 detected genes. We used the custom expression slot of the giotto object to add the normalized expression matrix as explained in the original paper [(Codeluppi et al. 2018)](https://paperpile.com/c/zehMrt/PbM8t). To cluster cells, PCA was performed on all genes with *runPCA* using the custom normalized expression matrix and parameter scale_unit = F. This step was followed by nonlinear dimensionality reduction using the *runUMAP* command on the first 31 identified PCs. After creating a sNN network with *createNearestNetwork* on the same 31 PCs, we then used *doLeidenCluster* to perform Leiden-clustering with resolution 0.09. This resulted in both moderate and many small sized clusters. We merged similar small sized clusters in a data-driven manner with the commands *getClusterSimilarity* and *mergeClusters* with parameters “max_group_size = 30, force_min_group_size = 25 and min_cor_score = 0.7”. To annotate each cell cluster, we visualized its gene expression pattern by using the command *plotMetaDataHeatmap* and compared with the annotations from the original paper. The two sets of annotations matched well. As a result, we identified oligodendrocyte precursors, COP oligodendrocytes, NF oligodendrocytes, mature oligodendrocytes, Flt+ or Apln+ endothelial cells, ependymal cells, Gfap+ astrocytes, Mfge8+ astrocytes, microglia, hippocampus, Crhbp+ interneurons, Cnr1+ interneurons, CP interneurons, Vip interneurons, Crh interneurons, vascular smooth muscle cell, perivascular macrophages (PVM), pericytes, choroid plexus and layer enriched excitatory neurons (L2/3/5, L3/4, L5 and L6). To compare the cell-type/cell-type interaction patterns, we first reduced and filtered the cell types of both datasets to oligodendrocytes, endothelial cells, astrocytes, L2/3 eNeurons, L4 eNeurons, L5 eNeurons, L6 eNeurons, iNeurons, microglia and mural cells. Then for both datasets the function *createSpatialNetwork* was used to create a spatial network as input for *cellProximityEnrichment.* The results of the cell-type/cell-type interaction analyses were visualized with the function *cellProximityHeatmap*.

*t-cyCIF human pancreatic ductal adenocarcinoma (PDAC)*

The t-cyCIF PDAC dataset and accompanying information was downloaded from <http://lincs.hms.harvard.edu/lin-elife-2018/> in companion with [(Lin et al. 2018)](https://paperpile.com/c/zehMrt/yVGrH), which was used to create figure 7 and 8 in the original publication [(Lin et al. 2018)](https://paperpile.com/c/zehMrt/yVGrH). As the input expression matrix we used all protein stainings, excluding Hoechst, MitoTracker, A555 and HCS red stainings, which mark the DNA, mitochondria, Golgi apparatus and cytoplasm respectively. The raw data were normalized using the command *normalizeGiotto* with parameter scalefactor = 10000. PCA was performed on the full protein expression matrix using *runPCA* with the default settings and further followed by non-linear dimension reduction with UMAP on the first 14 PCs. These 14 PCs were then used as input to create a sNN network with *createNearestNetwork* and parameter k = 20. Community based clusters were subsequently detected with Leiden clustering using 0.2 as the resolution parameter for the *doLeidenCluster* command. This results in a total of 13 clusters that were annotated based on the dominant expression patterns of the proteins.

*Visium mouse kidney*

The Visium data for mouse kidney coronal section was collected from 10X Genomics website (<https://www.10xgenomics.com/>) and the raw matrix was extracted with the function *get10Xmatrix.* This gene expression matrix together with the spatial locations of spots were used to create a giotto object with *createGiottoObject*. The *subsetGiotto* function was used to retain only those spots that had been annotated as overlapping with tissue based on the provided metadata (column 2 = 1). Low quality spots and low expressed genes were removed by using *filterGiotto* and parameter setting: “expression_threshold = 1, gene_det_in_min_cells = 50, min_det_genes_per_cell = 1000”. As a result, 7 cells and 6360 genes were removed from further analysis. The filtered expression matrix was subsequently normalized by using *normalizeGiotto*. To cluster spots highly variable genes were first detected by using *calculateHVG* and those highly variable genes that were also detected in > 4% of cells with a minimum log-normalized expression > 0.5 were used to perform PCA analysis by using *runPCA*. Next, nonlinear dimensionality reduction was achieved by applying the *runUMAP* function to the first 10 PCs. The same 10 PCs were also used to create a sNN network with *createNearestNetwork* and parameter k = 15. Finally, network communities representing clusters of spots were identified with Leiden clustering using *doLeidenCluster* and parameter resolution = 0.4. Spatial co-expression modules were detected with the function *detectSpatialCorGenes.* This function used as input a Delaunay spatial network created with *createDelaunayNetwork* and the top 500 spatial gens from the function *binSpect* with parameter ‘bin_method = ‘kmeans’, 8 clusters were identified using hierarchical clustering with the function *clusterSpatialCorGenes* and these results were visualized in using a heatmap with *heatmSpatialCorGenes.* To visualize metagenes that correspond to the identified co-expression modules we first created metagenes with the function *createMetagenes* followed by *spatCellPlot* for visualization in the spatial physical space. By cross-referencing this information with that of spatially enriched single-cell RNA-seq data [(Ransick et al. 2019)](https://paperpile.com/c/zehMrt/s32p) we could discriminate the different known mouse kidney structures. Metagene 4 (green) corresponded to the inner medulla, metagene 1 (magenta) corresponded to the inner and outer medulla, metagene 3 (blue) and 6 (grey) corresponded to the outer medulla, metagene 5 (brown) corresponded to the outer medulla and cortex, metagene 2 (orange) corresponded to the cortex alone and finally metagene 8 (yellow) and 7 (purple) were specific to the deep medullary epithelium of pelvis and kidney surrounding adipose tissue, respectively.

*Visium mouse brain*

The Visium data for the mouse brain coronal section was collected from 10X Genomics website (https://www.10xgenomics.com/) and the raw matrix was extracted with the function *get10Xmatrix*. The *subsetGiotto* function was used to retain only those spots that had been annotated as overlapping with tissue based on the provided metadata (column 2 = 1). Then, we filtered out low quality genes and spots by using *filterGiotto* with parameter: expression_threshold = 1, gene_det_in_min_cells = 50, min_det_genes_per_cell = 1000. The raw expression matrix was subsequently normalized using normalizeGiotto with default settings. To comprehensively perform cell-type enrichment analysis in Visium data of mouse brain, we collect cell type marker genes reported from [(Zeisel et al. 2018)](https://paperpile.com/c/zehMrt/vniY7) and then aggregated them based on taxonomy. The taxonomy marker genes were used for enrichment analysis using the PAGE method by *runSpatialEnrich* with parameter: enrich_method = 'PAGE'.

*merFISH mouse hypothalamic preoptic region*

The merFISH dataset for the mouse hypothalamic preoptic region and accompanying annotations were obtained from (<https://science.sciencemag.org/content/362/6416/eaau5324>) in companion with [(Moffitt et al. 2018)](https://paperpile.com/c/zehMrt/MXXjf). The cell locations and gene expression values from all 12 provided slices from the first mouse were used to create a giotto object with *createGiottoObject*. The gene expression matrix was then normalized with *normalizeGiotto* with the parameter ‘scalefactor = 10000’. All genes were used to perform PCA with *runPCA* and the first 8 PC’s were subsequently used to perform further nonlinear dimension reduction with UMAP using *runUMAP*. Next, a sNN network was created based on the same first 8 PCs using the *createNearestNetwork* function with parameter ‘k = 15’, followed by network community detection with the Leiden algorithm using *doLeidenCluster* with parameter ‘resolution = 0.2’. In total we identified 9 clusters which were annotated for cell type based on their displayed gene expression patterns visualized with *plotMetaDataHeatmap*. This resulted in the identification of excitatory neurons (*Slc17a6*), inhibitory neurons (*Gad1*), astrocytes (Aqp4), ependymal cells (*Cd24a*), endothelial cells (*Fn1*), microglia (*Selplg*), immature (*Pdgfra*) and mature (*Mbp*) oligodendrocytes and one ambiguous group of cells, which is similar to the original publication [(Moffitt et al. 2018)](https://paperpile.com/c/zehMrt/MXXjf). In general, the two sets of annotations were in good agreement, with the only exception that many cells annotated as ‘ambiguous’ in the original paper were re-assigned by Giotto to various cell types. Of these ambiguous cells, a significant fraction was assigned to the mature oligodendrocyte cluster and showed very similar gene expression profiles. After removing the ambiguous cells from comparison, the overall agreement between the two sets of annotations was high (adjusted rand index = 0.7).

*STARmap mouse visual cortex*

The STARmap mouse visual cortex data was downloaded from <https://www.starmapresources.com/data> in companion with [(Wang et al. 2018)](https://paperpile.com/c/zehMrt/4CRVN). The gene expression matrix together with the spatial locations of cells were used to create a giotto object with *createGiottoObject*. Low quality cells and lowly expressed genes were removed by using *filterGiotto* and parameters: ‘expression_threshold = 1, gene_det_in_min_cells = 20000, min_det_genes_per_cell = 20’, which resulted in the removal of 757 cells. All genes were retained after the filtering step. The filtered raw expression matrix was subsequently normalized with *normalizeGiotto*. To cluster cells, the highly variable genes were detected with *calculateHVG* with the method ‘cov_groups’, and filtered by ‘zscore_threshold = 0.5’ and ‘nr_expression_groups = 3’. The resulting genes were used to perform PCA analysis with *runPCA*. Further nonlinear dimension reduction was applied with UMAP using the *runUMAP* function on the first 8 PCs. The same 8 PCs were also used to create a sNN network with *createNearestNetwork* and parameter ‘k = 15’. Finally, network communities representing clusters of similar cells were identified with Leiden clustering using *doLeidenCluster* and parameter resolution = 0.2. To create 2D slices of the 3D STARmap dataset we first filtered out the top and bottom 5% of the data along the z-direction. For the remaining part of the 3D volume, 7 consecutive virtual slices with thickness 10 *u*m along the z-direction were created. Then 2D cell proximity analysis was applied on the x and y coordinates of each slice using the *cellProximityEnrichment* function. The results were then combined to generate a consensus result for the whole volume (2D result). The 3D analysis for the whole volume was done using all the 3 coordinates (x, y and z) from the whole volume instead.

*CODEX mouse spleen*

The CODEX data was downloaded from Mendeley (<http://dx.doi.org/10.17632/zjnpwh8m5b.1#file-e51e833d-1317-454b-b4d7-30850ec4bb78>) in companion with [(Goltsev et al. 2018)](https://paperpile.com/c/zehMrt/1fVKq). Tile specific X and Y coordinates were converted to global coordinates for each spleen tissue slice using *stitchCoordinate*. 83,787 cells from sample "BALBc-3” were selected for the analysis. The gene expression matrix together with the spatial locations of cells were used to create a giotto object with *createGiottoObject*. The "dirt", "noid" and "capsule" cells based on the provided "Imaging_Phenotype_annotation" were excluded. Low quality cells and lowly expressed genes were removed by using *filterGiotto* and parameter setting: 'expression_threshold = 1, gene_det_in_min_cells = 10, min_det_genes_per_cell = 2'. 80017 cells and 30 genes passed the filtering step. The raw expression matrix was subsequently normalized with *normalizeGiotto* with the option "scale_cells=TRUE". To cluster cells, all 30 genes were used to perform PCA with *runPCA*. Further nonlinear dimension reduction was applied with UMAP using the *runUMAP* function on the first 14 PCs. The same 14 PCs were also used to create a sNN network with *createNearestNetwork* and parameter k = 20. Finally, cell clusters were identified with Leiden clustering using *doLeidenCluster* and parameter resolution = 0.5. We zoomed in to regions with distinct cell-type distribution patterns, and identified zones enriched with distinct cell type distribution patterns (**Fig. S4G-I**). For example, Zone 1 was highly enriched with CD8(+) T cells, whereas Zone 2 was enriched with erythroblasts and F4/80 macrophages.

*MIBI human triple-negative breast cancer samples*

MIBI data from 40 triple-negative breast cancer (TNBC) patients were obtained from [(Keren et al. 2018)](https://paperpile.com/c/zehMrt/GGkVQ). We used the processed and normalized data matrix, where the values were z-scores. The dataset had 41 protein markers, from which we filtered all elemental markers (Au, Na, K, etc) and histone marks which resulted in a set of 34 proteins for further analysis. We clustered the data by using k-means (with *doKmeans* function in Giotto, k=20, nstart=100). Two of the resulting clusters (corresponding to Keratin, and B/T cell cluster) were subject to subclustering analysis for refinement, which was facilitated by the *subsetGiotto* function. As a result, the Keratin cell cluster was split into three subclusters: Keratin, EGFR, and beta-catenin, whereas the B/T cell cluster was split into a B cell subcluster and a CD45+ T cell subcluster (merged with the “Other.T'' cell group). For the cell neighborhood analysis, we created a spatial network with the number of nearest neighbors set to 5, and then applied *cellProximityEnrichment* (number of simulations = 1000) and *cellProximityNetwork* (remove_self_edges = T, only_show_enrichment_edges = T).

In patients 4 and 5, the Keratin-marked epithelial cells and immune cells were well segregated from each other. Patients 10 and 17 featured a rather mixed environment between T cells, Keratin, and Ki67 cancer cells. Additionally, Patients 17 and 35 contained a distinctive B cell cluster, and extensive B cell-T cell interactions caused by the B-cell colocalization in their spatial profiles. We found a common colocalization pattern of fibroblasts (SMA/vimentin) and monocytes across most of the networks. The crosstalk between fibroblasts and immune monocytes may indicate the ability for cancer associated fibroblast to modulate the immune environment, possibly affecting the anti-tumor immune response of effector cells [(Liu et al. 2019)](https://paperpile.com/c/zehMrt/MhAm7). Consistent with this finding, previous studies have indicated the recruitment of blood monocytes by cancer associated fibroblasts into the tumor tissue [(Silzle et al. 2003)](https://paperpile.com/c/zehMrt/jkWJN), which has immunosuppressive implication [(Gok Yavuz et al. 2019)](https://paperpile.com/c/zehMrt/U3fbE).

*Slide-seq mouse cerebellum*

Slide-seq data from mouse cerebellum was obtained from Broad Single Cell Portal (<http://singlecell.broadinstitute.org/single_cell>) in companion with [(Rodriques et al. 2019)](https://paperpile.com/c/zehMrt/a7aZo). For cell-type enrichment analysis, a scRNA-seq dataset was obtained from [http://mousebrain.org](http://mousebrain.org/) under “Tissues” tab and then under “Cerebellum”. Cell types and associated gene signatures were identified by Leiden clustering of the scRNA-seq dataset with default setting in Giotto. We next computed a ranking of cell type specific genes for each cell type (with *makeSignMatrixRank* function which generates a rank matrix). On the Slide-seq end, we first filtered genes and cells based on minimal gene and cell coverages (20 genes and 20 cells). Cell type enrichment within each Slide-seq bead was then performed, using individual bead’s normalized expression counts and the cell type gene signature rank matrix (of scRNA-seq). This was achieved by using the *runRankEnrich* function (setting sign_matrix=rank_matrix, reverse_log_scale = FALSE, rbp_p = 0.99, num_agg=100 and ties.method=”random”). Enrichment analysis results were displayed by using the *spatCellPlot()* function.

**Benchmarking results**

*Comparison of marker gene detection methods*

To systematically compare and evaluate the differences between the 3 provided marker gene detection methods, we computed the top 20 marker genes and their overlap for each identified cell type in the seqFISH+ somatosensory cortex dataset with all 3 methods provided in Giotto Analyzer, i.e. Gini, Mast [(Finak et al. 2015)](https://paperpile.com/c/zehMrt/r8Dt0) and Scran [(Lun et al. 2017)](https://paperpile.com/c/zehMrt/k7MWH) (**Fig. S2A**). Here we introduce the Gini method to identify marker genes based on the assumption that the perfect marker gene would only be expressed in one identified cluster and hence would display a strong unequal distribution of its transcripts among all the identified clusters (**Fig. S2B**). Such inequality can be measured by the Gini coefficient and has been previously used to identify rare cell types at the single-cell level [(Jiang et al. 2016)](https://paperpile.com/c/zehMrt/XKlKW) (see **Methods**).To evaluate the run time for each method, we used the *tictoc* R package on a standard MacOS computer with 16G memory and 4 Intel Core i7 CPUs. This showed that both Gini and Scran were considerably faster as compared to Mast (**Fig. S2C**). To evaluate if all methods could detect known cell type markers we compared the unique and overlapping genes for all cell types. This demonstrated that known cell type markers were almost always retrieved by all methods, such as *Cldn5* and *Pltp* for endothelial cells or *Sox10* and *Fa2h* for oligodendrocytes (**Fig. S2D**). However, we also noticed a significant number of genes that were only identified by one method. To evaluate how these genes differ, we assessed the specificity and sensitivity of those genes for their respective cell type cluster.

Therefore, we converted the expression matrix into a binarized expression matrix by applying kmeans (k = 2) on all the rows (= genes). These binarized values were then subsequently used to calculate the sensitivity and specificity scores for the genes that were only prioritized by one, but not by any other, method. This analysis showed that for both endothelial cells and oligodendrocytes the Gini-specific genes were more specific, but displayed reduced sensitivity as marker genes for that cell type (**Fig. S2E**). In fact, this observation was generalizable for all clusters, such that genes prioritized only by Scran are more sensitive, while genes prioritized only by Gini are more specific (**Fig. S2F**). This increased specificity can also be visually observed by plotting the top prioritized genes for each method using a violin plot (**Fig. S2G**).

*Cell proximity changes for 3D data vs 2D slices*

We performed cell-cell proximity analysis for both the 2D slices and the 3D volume of the STARmap dataset (**Fig. S3E**) and noticed that in general the 2D and 3D analysis were highly correlated (**Fig. S3F**). However, the percent-deviation of the 2D result from the 3D one tended to be negatively correlated with the absolute proximity score (**Fig. S3F**), indicating the potential limitation of the accuracy of 2D analysis as compared to 3D analysis. Of note, the overall high similarity is not surprising given that the z-axis for the STARmap dataset is relatively small (~30-fold) compared to the x and y axes and that the structure of the mouse visual cortex does not change that much in this direction at this short distance. We anticipate that, with more complex or bigger tissues, the benefit of having 3D information will steadily increase.

*Estimation of cell-type distributions*

Giotto implements three enrichment analysis methods for estimating cell-type distributions: PAGE, RANK, and the hyper-geometric test. In addition, cell-type distribution can also be estimated by using a simple approach based on Spearman correlation and by using a recently published method called RCTD [(Cable et al. 2020)](https://paperpile.com/c/zehMrt/lUrf). The RCTD program was run by using the function create.RCTD (setting UMI_min=10), followed by the function run.RCTD (setting doublet_mode = TRUE) on gene expression matrices in the raw counts form.

To systematically compare the performance, each method was applied to the coarse-gridded seqFISH+ dataset (see **Methods** for details) and compared to the ground-truth. The accuracy of each method was quantified by the area under curve (AUC) score, which was evaluated by using “pROC” package in R for each cell type. For this simulated dataset, PAGE, RANK, and RCTD all had high accuracy (**Fig. S6A**) and were robust to changes in number of transcripts (UMIs) (**Fig. S6B**). However, RCTD ran much slower (**Fig. S6C**), making it difficult to analyze large datasets.

For real dataset analysis, the batch effect is a well-known technical artifact that may lead to erroneous conclusions. To assess the potential impact of the batch effect on cell-type distribution estimation, we applied both RANK (as a representative example of our enrichment analysis) and RCTD to analyze a published Slide-seq dataset. Since the ground-truth was unknown, we compared the overlap between the outcome from these two methods and quantified the degree of agreement by using the Pearson correlation coefficients. The resulting patterns were quite similar (**Fig. S7B, C**).

*Comparison of spatial gene detection methods*

To systematically compare and evaluate the difference between the 5 provided spatial gene detection methods, we applied each method to both seqFISH+ somatosensory cortex and Visium kidney dataset using the default settings. For each method the top 1000 genes were selected based on the ranking provided by each method and used to calculate pairwise correlation (Spearman) coefficient of the overlapping genes. This illustrated that BinSpect-kmeans, BinSpect-Rank, SPARK [(Sun et al. 2020)](https://paperpile.com/c/zehMrt/AoEY) and SpatialDE [(Svensson et al. 2018)](https://paperpile.com/c/zehMrt/GxKki) were similar in terms of ranking, while trendsceek [(Edsgärd et al. 2018)](https://paperpile.com/c/zehMrt/3OVth) resulted in a more different ranking for both datasets (**Fig. S8D-E**). A similar conclusion was seen in terms of the number of overlapping genes (**Fig. S8F-G**). Here, trendsceek returned the highest number of unique spatial genes. On the other hand, a large number of genes were detected in 3 out of 5 methods. This observation was also seen in both datasets, although more consistent results were obtained for the Visium kidney dataset. Finally, we evaluated the computing time with the built-in R command *system.time()* using an MacOS computer with 32G memory and 8 CPUs and noted that for both datasets the BinSpect methods were significantly faster compared to SPARK, SpatialDE and trendsceek (**Fig. S8H-I**). To evaluate how efficient each method was at detecting known spatial gene expression patterns, we inserted each individual simulated gene expression profile (see **Methods**) back into the original dataset – ensuring to stay as close to the original and real gene expression space as possible - and then ran all the methods with their default parameters. Here, the objective was solely to evaluate if the known spatial gene expression pattern could be retrieved or not (given a fixed adjusted p-value threshold = 0.01). After completing this for all 4200 simulated patterns we then plotted the power (fraction of detected) vs the probability (proxy for how strong the simulated pattern is). We noticed that while almost all methods did not identify any spatial genes with Pr = 0.5 (random patterns) and could retrieve most patterns when Pr = 1 (strongest patterns are generated), there was a discrepancy when the probability was reduced (introduction of noise) (**Fig. S8J**). It is possible to further improve the performance by using more sophisticated methods to calibrate p-values, but this is beyond the scope of this paper.

*Effect of spatial network parameters on spatial co-expression patterns.*

To assess how the number of nearest spatial neighbors (k parameter) or radial distance from the cell centroid (distance parameter) might affect the identification of spatial genes and co-expression networks, a quantitative and qualitative robustness analysis was performed. Therefore, a range of spatial networks with different k (4, 8, 16, 32, 64) or distance (200, 300, 400, 500, 600) were generated (**Fig. S10A**) and used to examine how this would impact the top 8 spatial co-expression modules within the Visium Kidney dataset (see Datasets). First, spatial genes were calculated using the BinSpect-Kmeans method for all the spatial networks. For each condition the identified spatial genes were ranked based on the returned odds-ratio multiplied by -log(p.value) and compared using pairwise correlations with the other conditions. This showed that the rank of spatial genes was insensitive to the parameter k and distance (**Fig. S10B**). Next, to evaluate whether the co-expression modules consist of the same genes, all identified spatial genes (p-value < 0.05, odds-ratio > 1.5 and hub-ratio > 0.05) were selected and used to create spatial co-expression modules (hierarchical clustering with k = 8) Based on pairwise calculated adjusted rand index scores, we observed that many genes were found in the same co-expression modules across conditions (**Fig. S10C**). Nevertheless, to see if spatial network specific differences would lead to different global co-expression modules, we selected the top 100 spatial genes from each spatial network condition and visualized the co-expression modules as metagenes for the most different cases (k = 2 or 64 and distance = 200 or 600). This qualitative assessment showed that almost all global patterns could be retrieved with each used spatial network (**Fig. S10D**). In addition, those patterns that were not detected in one specific condition, could be retrieved by increased the number of spatial co-expression modules (data not shown).

*Effect of spatial network parameter k on identification of interacting cell types.*

To evaluate how enrichment or depletion of interacting cell types depends on the number of cell neighbors (parameter k), we first created multiple spatial networks using a range of k’s that likely reflect the immediate neighbourhood of a cell (k = 3, 4, 5, 6, 7 or 8) on the seqFISH+ mouse somatosensory cortex dataset and then assessed cell-type/cell-type interacting patterns by evaluating the enrichment of the frequency that they are proximal to each other (**Fig. S11A**). From all the 78 pairs of cell types that were assessed, 14 pairs (17.9%) were significantly enriched or depleted (FDR < 0.05) in all 6 conditions, 6 pairs (7.7 %) in 5 out 6 conditions, 2 pairs (2.6%) in 4 out of 6 conditions, 4 pairs (5.1%) in 3 out of 6 conditions, the remainder of cell pairs (52 or 66.7%) were never significantly enriched or depleted. This indicates that the majority of significantly enriched or depleted pair of cell types interactions were identified in all (53.8%) or nearly all of the different k parameter values.

*Effect of spatial network parameter k on spatial ligand-receptor pairing.*

To test the effect of the spatial network parameter k on the spatial ligand-receptor pairing we tested 3 different values of k (4, 5 and 6). After ranking the ligand-receptor pairs based on a significance score (S), we calculated the correlation of the S on the top 200 identified ligand-receptor pairs in the 3 different conditions over all cell type pairs.

$$S=\left| FC*-log10\left( adjustedpvalue \right) \right|$$

This result indicated that in general the top prioritized ligand-receptor pairs are similar in all conditions (**Fig. S11C**).

**References**

[Cable DM, Murray E, Zou LS, et al (2020) Robust decomposition of cell type mixtures in spatial transcriptomics. bioRxiv](http://paperpile.com/b/zehMrt/lUrf)

[Codeluppi S, Borm LE, Zeisel A, et al (2018) Spatial organization of the somatosensory cortex revealed by osmFISH. Nat Methods 15:932–935](http://paperpile.com/b/zehMrt/PbM8t)

[Edsgärd D, Johnsson P, Sandberg R (2018) Identification of spatial expression trends in single-cell gene expression data. Nat Methods 15:339–342](http://paperpile.com/b/zehMrt/3OVth)

[Eng C-HL, Lawson M, Zhu Q, et al (2019) Transcriptome-scale super-resolved imaging in tissues by RNA seqFISH+. Nature 568:235–239](http://paperpile.com/b/zehMrt/fTsh)

[Finak G, McDavid A, Yajima M, et al (2015) MAST: a flexible statistical framework for assessing transcriptional changes and characterizing heterogeneity in single-cell RNA sequencing data. Genome Biol 16:278](http://paperpile.com/b/zehMrt/r8Dt0)

[Gok Yavuz B, Gunaydin G, Gedik ME, et al (2019) Cancer associated fibroblasts sculpt tumour microenvironment by recruiting monocytes and inducing immunosuppressive PD-1+ TAMs. Sci Rep 9:3172](http://paperpile.com/b/zehMrt/U3fbE)

[Goltsev Y, Samusik N, Kennedy-Darling J, et al (2018) Deep Profiling of Mouse Splenic Architecture with CODEX Multiplexed Imaging. Cell 174:968–981.e15](http://paperpile.com/b/zehMrt/1fVKq)

[Jiang L, Chen H, Pinello L, Yuan G-C (2016) GiniClust: detecting rare cell types from single-cell gene expression data with Gini index. Genome Biol 17:144](http://paperpile.com/b/zehMrt/XKlKW)

[Keren L, Bosse M, Marquez D, et al (2018) A Structured Tumor-Immune Microenvironment in Triple Negative Breast Cancer Revealed by Multiplexed Ion Beam Imaging. Cell 174:1373–1387.e19](http://paperpile.com/b/zehMrt/GGkVQ)

[Lin J-R, Izar B, Wang S, et al (2018) Highly multiplexed immunofluorescence imaging of human tissues and tumors using t-CyCIF and conventional optical microscopes. Elife 7.: https://doi.org/](http://paperpile.com/b/zehMrt/yVGrH)[10.7554/eLife.31657](http://dx.doi.org/10.7554/eLife.31657)

[Liu T, Han C, Wang S, et al (2019) Cancer-associated fibroblasts: an emerging target of anti-cancer immunotherapy. J Hematol Oncol 12:86](http://paperpile.com/b/zehMrt/MhAm7)

[Lun A, Bach K, Kim JK, et al (2017) Package “scran”](http://paperpile.com/b/zehMrt/k7MWH)

[Moffitt JR, Bambah-Mukku D, Eichhorn SW, et al (2018) Molecular, spatial, and functional single-cell profiling of the hypothalamic preoptic region. Science 362.: https://doi.org/](http://paperpile.com/b/zehMrt/MXXjf)[10.1126/science.aau5324](http://dx.doi.org/10.1126/science.aau5324)

[Ransick A, Lindström NO, Liu J, et al (2019) Single-Cell Profiling Reveals Sex, Lineage, and Regional Diversity in the Mouse Kidney. Dev Cell 51:399–413.e7](http://paperpile.com/b/zehMrt/s32p)

[Rodriques SG, Stickels RR, Goeva A, et al (2019) Slide-seq: A scalable technology for measuring genome-wide expression at high spatial resolution. Science 363:1463–1467](http://paperpile.com/b/zehMrt/a7aZo)

[Silzle T, Kreutz M, Dobler MA, et al (2003) Tumor-associated fibroblasts recruit blood monocytes into tumor tissue. Eur J Immunol 33:1311–1320](http://paperpile.com/b/zehMrt/jkWJN)

[Sun S, Zhu J, Zhou X (2020) Statistical analysis of spatial expression patterns for spatially resolved transcriptomic studies. Nat Methods 17:193–200](http://paperpile.com/b/zehMrt/AoEY)

[Svensson V, Teichmann SA, Stegle O (2018) SpatialDE: identification of spatially variable genes. Nat Methods 15:343–346](http://paperpile.com/b/zehMrt/GxKki)

[Wang X, Allen WE, Wright MA, et al (2018) Three-dimensional intact-tissue sequencing of single-cell transcriptional states. Science 361.: https://doi.org/](http://paperpile.com/b/zehMrt/4CRVN)[10.1126/science.aat5691](http://dx.doi.org/10.1126/science.aat5691)

[Zeisel A, Hochgerner H, Lönnerberg P, et al (2018) Molecular Architecture of the Mouse Nervous System. Cell 174:999–1014.e22](http://paperpile.com/b/zehMrt/vniY7)
