# Supplementary material for: Giotto: a toolbox for integrative analysis and visualization of spatial expression data
Source: Genome Biol. 2021 Mar 8;22:78. doi: 10.1186/s13059-021-02286-2 (PMC7938609; doi:10.1186/s13059-021-02286-2)
Supplement: Supplementary file 5 — Additional file 5. Review history. [file 13059_2021_2286_MOESM5_ESM.docx]

Review History

This manuscript was previously reviewed at another journal and our reviewer primarily assessed the previous comments.

**First round of review**

**Reviewer 1**

**Were you able to assess all statistics in the manuscript, including the appropriateness of statistical tests used?**

Yes

**Comments to author:**

This manuscript describes a new software tool, Giotto, for analysis and visualization of spatial expression data. Giotto contains a data analysis module for characterizing cell-type distribution, spatially coherent gene expression patterns, and interactions between each cell and its surrounding neighbors. Giotto also contains a data visualization module that allows users to interactively visualize the gene expression data, analysis outputs, and additional imaging features, thereby providing a user-friendly workspace to explore multiple modalities of information for biological investigation.

The manuscript is well written, and Giotto is a very useful toolbox for the spatial gene expression field. Several methods implemented in Giotto are also novel and show advantages as compared to existing approaches. For example, one of the new methods in Giotto is BinSpect, which is used for detecting spatial co-expression patterns and is faster than many existing methods. For BinSpect, however, I was wondering whether it is also able to produce calibrated p-values under the null as the recent method SPARK. A key feature of SPARK is that it can produce calibrated p-values, which are important for identifying co-expression patterns at the transcriptome-wide threshold. It would be useful to show some qq-plots on -log10(p-values) from BinSpect under the null, to see if its p-values are also as calibrated as SPARK. It would be important to brought up the p-value calibration issue in the main text. But I feel this is a relatively minor issue and Giotto will be a useful toolbox regardless whether the BinSpect p-values are calibrated or not.

**Authors Response**

**Point-by-point responses to the reviewers’ comments:**

Reviewer #1:

This manuscript describes a new software tool, Giotto, for analysis and visualization of spatial expression data. Giotto contains a data analysis module for characterizing cell-type distribution, spatially coherent gene expression patterns, and interactions between each cell and its surrounding neighbors. Giotto also contains a data visualization module that allows users to interactively visualize the gene expression data, analysis outputs, and additional imaging features, thereby providing a user-friendly workspace to explore multiple modalities of information for biological investigation.

*RESPONSE: Thank you for highlighting several of the novelties that we present in Giotto and considering Giotto a user-friendly workspace for spatial data exploration, which is the primary goal of Giotto.*

The manuscript is well written, and Giotto is a very useful toolbox for the spatial gene expression field. Several methods implemented in Giotto are also novel and show advantages as compared to existing approaches. For example, one of the new methods in Giotto is BinSpect, which is used for detecting spatial co-expression patterns and is faster than many existing methods. For BinSpect, however, I was wondering whether it is also able to produce calibrated p-values under the null as the recent method SPARK. A key feature of SPARK is that it can produce calibrated p-values, which are important for identifying co-expression patterns at the transcriptome-wide threshold. It would be useful to show some qq-plots on -log10(p-values) from BinSpect under the null, to see if its p-values are also as calibrated as SPARK. It would be important to brought up the p-value calibration issue in the main text. But I feel this is a relatively minor issue and Giotto will be a useful toolbox regardless whether the BinSpect p-values are calibrated or not.

*RESPONSE: We thank the reviewer for this interesting suggestion. We simulated a null data by setting Pr = 0.5 using the coarse-grained seqFISH+ dataset as described in the Supplementary Note. The relationship between observed and expected p-value distribution is shown in the qqplot on the right. As the reviewer suspected, the p-values obtained from binSpect is not well-calibrated. Despite this limitation, our analysis results shown in Fig. S8J (attached below) indicate that setting a cutoff value at adjusted p-value = 0.01 is sufficient to remove false positives while maintaining high sensitivity. As such, we expect that the benefit of further improvement by p-value calibration is likely to be modest and it is beyond the scope of this paper. We added the following sentence in Supplementary Notes to acknowledge this point. “It is possible to further improve the performance by using more sophisticated methods to calibrate p-values, but this is beyond the scope of this paper.”*

*Fig. S8J. Spatial gene detection simulation results. Different simulated patterns are show on the left (green = pattern, red = remaining cell locations) and corresponding spatial gene detection results are show on the right. A loess smoothened curve is shown for each method depicting the average fraction of retrieved genes (y-axis) at different noise levels (x-axis). Noise is controlled by a probability parameter (Pr), with 0.5 = random pattern and 1 = strong pattern.*

*In addition, BinSpect provides additional information that allows a user to identify potential spurious spatial results in an explorative manner. This includes calculating the average expression of high expressing cells in the binarized network, calculating the number of high expressing cells and calculating how many hub cells can be identified. Hub cells are defined as high expressing cells in the binarized network which have a minimum (user defined) of other high expressing neighbor cells. Lastly to obtain potentially more robust results we have also implemented a multi-version of BinSpect (binSpectMulti) which automatically performs the same algorithm over a range of k-neighbor spatial networks and outputs an aggregated score.*
